# Transcriptomics in Human Challenge Models

**DOI:** 10.3389/fimmu.2017.01839

**Published:** 2017-12-18

**Authors:** Amber J. Barton, Jennifer Hill, Andrew J. Pollard, Christoph J. Blohmke

**Affiliations:** ^1^Oxford Vaccine Group, Department of Paediatrics, University of Oxford and the NIHR Oxford Biomedical Research Centre, Oxford, United Kingdom

**Keywords:** transcriptomics, vaccines, functional genomics, biomarkers, microarray, human challenge, expression, experimental infection

## Abstract

Human challenge models, in which volunteers are experimentally infected with a pathogen of interest, provide the opportunity to directly identify both natural and vaccine-induced correlates of protection. In this review, we highlight how the application of transcriptomics to human challenge studies allows for the identification of novel correlates and gives insight into the immunological pathways required to develop functional immunity. In malaria challenge trials for example, innate immune pathways appear to play a previously underappreciated role in conferring protective immunity. Transcriptomic analyses of samples obtained in human challenge studies can also deepen our understanding of the immune responses preceding symptom onset, allowing characterization of innate immunity and early gene signatures, which may influence disease outcome. Influenza challenge studies demonstrate that these gene signatures have diagnostic potential in the context of pandemics, in which presymptomatic diagnosis of at-risk individuals could allow early initiation of antiviral treatment and help limit transmission. Furthermore, gene expression analysis facilitates the identification of host factors contributing to disease susceptibility, such as *C4BPA* expression in enterotoxigenic *Escherichia coli* infection. Overall, these studies highlight the exceptional value of transcriptional data generated in human challenge trials and illustrate the broad impact molecular data analysis may have on global health through rational vaccine design and biomarker discovery.

## Introduction

Human challenge studies, the deliberate infection of volunteers with a pathogen of interest, have been used to interrogate disease pathogenesis and vaccine efficacy since the smallpox challenge of James Phipps by Edward Jenner in 1796 (1). Human challenge studies have facilitated exploration of many aspects of infectious diseases, ranging from factors affecting susceptibility (2) and basic immune mechanisms to diagnostic biomarkers (3) and vaccine-mediated protection (4). Since some of the early challenge studies, such as those led by Theodore Woodward (5) and Myron Levine (6–8), the number of measurable parameters has extended far beyond vaccine efficacy, clinical symptoms, and antibody titers, now routinely including detailed measurements of molecular parameters such as cytokines, gene expression, and pathogen load (9, 10).

Gene expression studies have become a widely used tool to interrogate human host responses to infection and immunological perturbation. The evolution of gene expression technologies has allowed changes in the expression of thousands of genes in the peripheral blood and tissues to be measured in response to vaccination or experimental infection. Starting in the 1990s with serial analyses of gene expression (11), two methods have come to dominate contemporary transcriptomics: microarrays and RNA sequencing (RNA-seq). Whereas microarrays measure the hybridization of fluorescently labeled transcripts (mRNA) to nucleotide probes on a bead chip, RNA-seq involves sequencing of each transcript followed by alignment to a reference genome. Although cheaper and less labor intensive, microarrays are now largely being superseded by RNA-seq (12).

**Figure 1:**
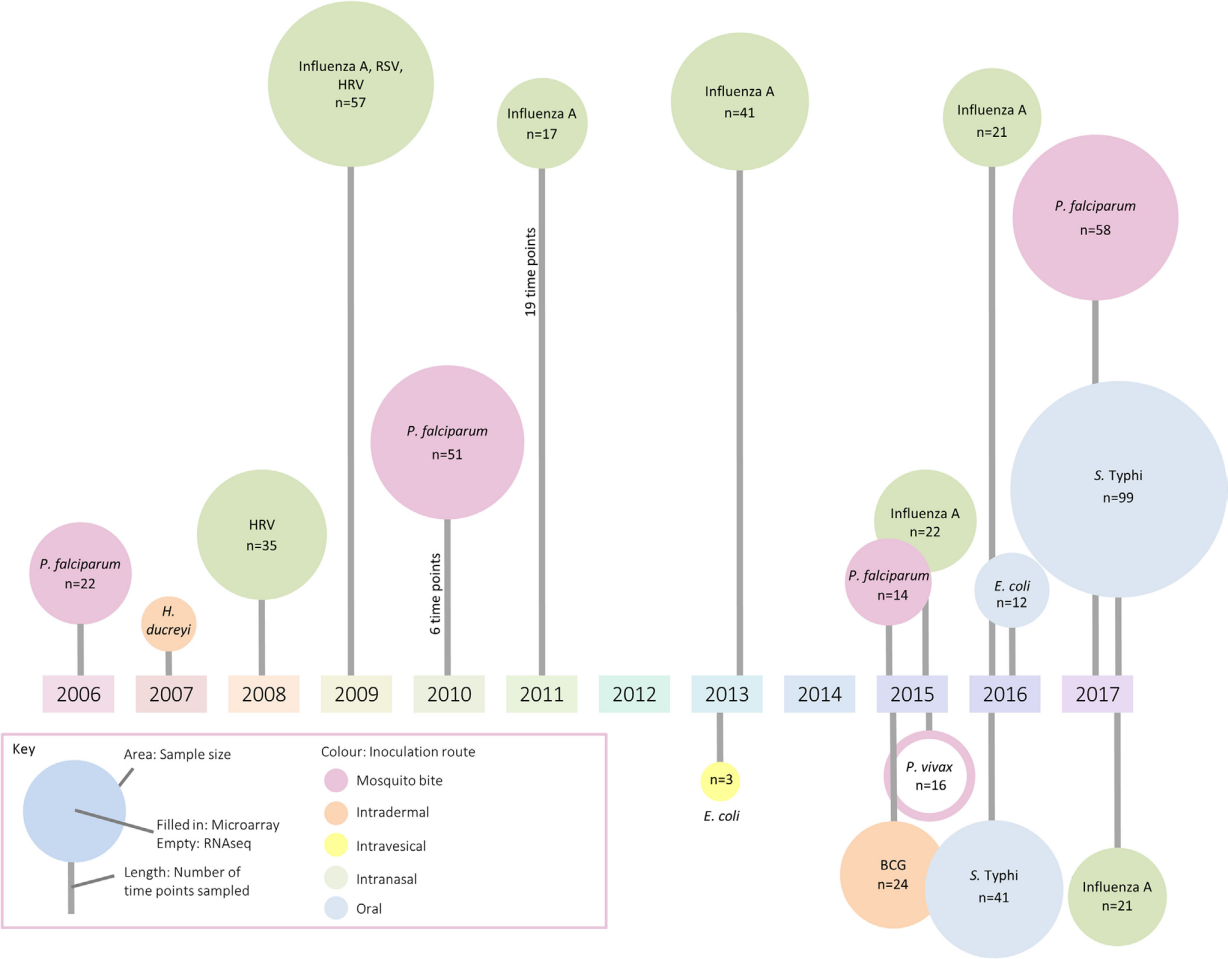
A summary of identified studies which have investigated changes in the transcriptome following controlled human infection. The area of each circle represents sample size, the color the inoculation route, and the line length indicates the number of samples taken for analysis.

Because technologies to measure gene transcription are sensitive methods to broadly assess responses using small amounts of blood (≤3 ml), these approaches have inevitably been applied to the field of controlled human infection models. These models provide the unique opportunity to follow the host transcriptional response from the resting state (prechallenge baseline) through overt clinical disease to convalescence. Although most gene expression studies to date have aimed to deepen understanding at the level of basic mechanisms and pathways, it is hoped the understanding of immune responses will facilitate applications ranging from rational vaccine design to identification of selective and specific prognostic and diagnostic biomarkers of infection. In this review, we summarize the application of transcriptomics to human challenge models, providing examples of work exploring (i) host factors affecting susceptibility to disease, (ii) host–pathogen interactions, (iii) factors associated with symptom severity, (iv) modulation of immune responses by vaccination, and (v) early exposure signatures with diagnostic potential. Although human challenge studies are being deployed with increasing frequency, only a handful of studies exploring the transcriptomic response were identified (summarized in Figure 1; Table S1 in Supplementary Material). The paucity of such publications indicates that there may be considerable missed opportunity for exploitation of these unique clinical models, in order to understand the biology of disease and the interventions used to prevent or treat them (13).

### Search Strategy

References were identified through Scopus using search terms (“expression signature” OR transcriptional OR signatures OR profiling OR “host response” OR “rna-seq” OR “RNA sequencing” OR “gene expression” OR transcriptome OR microarray OR “gene signature” OR illumina OR host OR profile OR profiling) AND (“human challenge” OR volunteers OR “experimental infection”). The names of known human challenge agents (13) were also searched in functional genomics databases ArrayExpress and Gene Expression Omnibus, filtering for human gene expression data. Relevant papers cited by the database were included.

## Host Factors Affecting Susceptibility

Challenge studies are well suited to analysis of host factors affecting susceptibility to infection, due to elimination of confounding factors such as variability between pathogen strains, or level and route of exposure to the pathogen. Although genome wide association studies (GWAS) are key in determining single nucleotide polymorphisms (SNPs) associated with susceptibility, the small number of subjects in challenge studies compared with studies of natural infection precludes identification of significantly associated genomic loci *via* this approach (14). However, the dramatic reduction in external variables afforded by human challenge studies, and the smaller number of genes assayed in microarrays compared with the millions of SNPs in GWAS, raises the possibility of identifying gene expression signatures present prior to experimental infection associated with susceptibility and protection. Such signatures may be influenced not only by genetic factors but also by the environment and epigenetic state, two factors with critical involvement in resistance and resilience. While the relationship between genetic variability, transcription, and disease susceptibility could be addressed by expression quantitative trait locus mapping (15), correlating genomic and transcriptomic data and applicable to sample sizes smaller than 100, to our knowledge this analysis has not yet been applied in the context of human challenge.

Studies examining the correlation between baseline characteristics (such as cell subset frequencies, antibody titer, or antibody functionality) with challenge outcome are numerous (16–19). In contrast, studies in which gene expression measured at baseline or shortly after challenge are correlated with clinical outcome are relatively few (2, 20). In a study by Yang et al., baseline gene expression was compared between equal numbers of asymptomatic individuals, and those who developed severe symptoms following oral challenge with enterotoxigenic *Escherichia coli* (ETEC) (2). Although there were only subtle differences in gene expression at this time point, several factors which plausibly might affect disease outcome were identified. For example, probes associated with presentation of antigen by major histocompatibility complex (MHC) molecules were enriched in those who did not develop infection, while baseline expression of complement inhibitor *C4BPA* was significantly higher in those who did. In another study, participants underwent multiple consecutive challenges with the Gram-negative bacterium *Haemophilus ducreyi*, responsible for the sexually transmitted infection chancroid (20). Comparison of the transcriptome from lesions sampled 48 h after inoculation between study subjects who consistently resolved infection and those who formed infected pustules supported the hypothesis that the balance of dendritic cell (DC) phenotypes at the inoculation site is of critical importance. In particular, signatures suggested that the presence or absence of regulatory DCs is crucial in determining whether lesion formation will occur. *In vitro* infection of monocyte-derived DCs from participants combined with subsequent gene expression analysis mirrored these findings, supporting the baseline distribution of host DC phenotypes as a major susceptibility factor. Interestingly, both studies demonstrated that either components of the innate immune system involved in the earliest contact with the pathogen or aspects linking the innate and the adaptive immune system played an important role. Overall, the lack of studies examining whether the host transcriptome at time of challenge is predictive of disease outcome represents a major gap in the literature; however, the accumulation of data sets from challenge studies presents an opportunity to further interrogate baseline status in the context of disease outcome following infection.

## Elucidating Host–Pathogen Interactions

**Figure 2:**
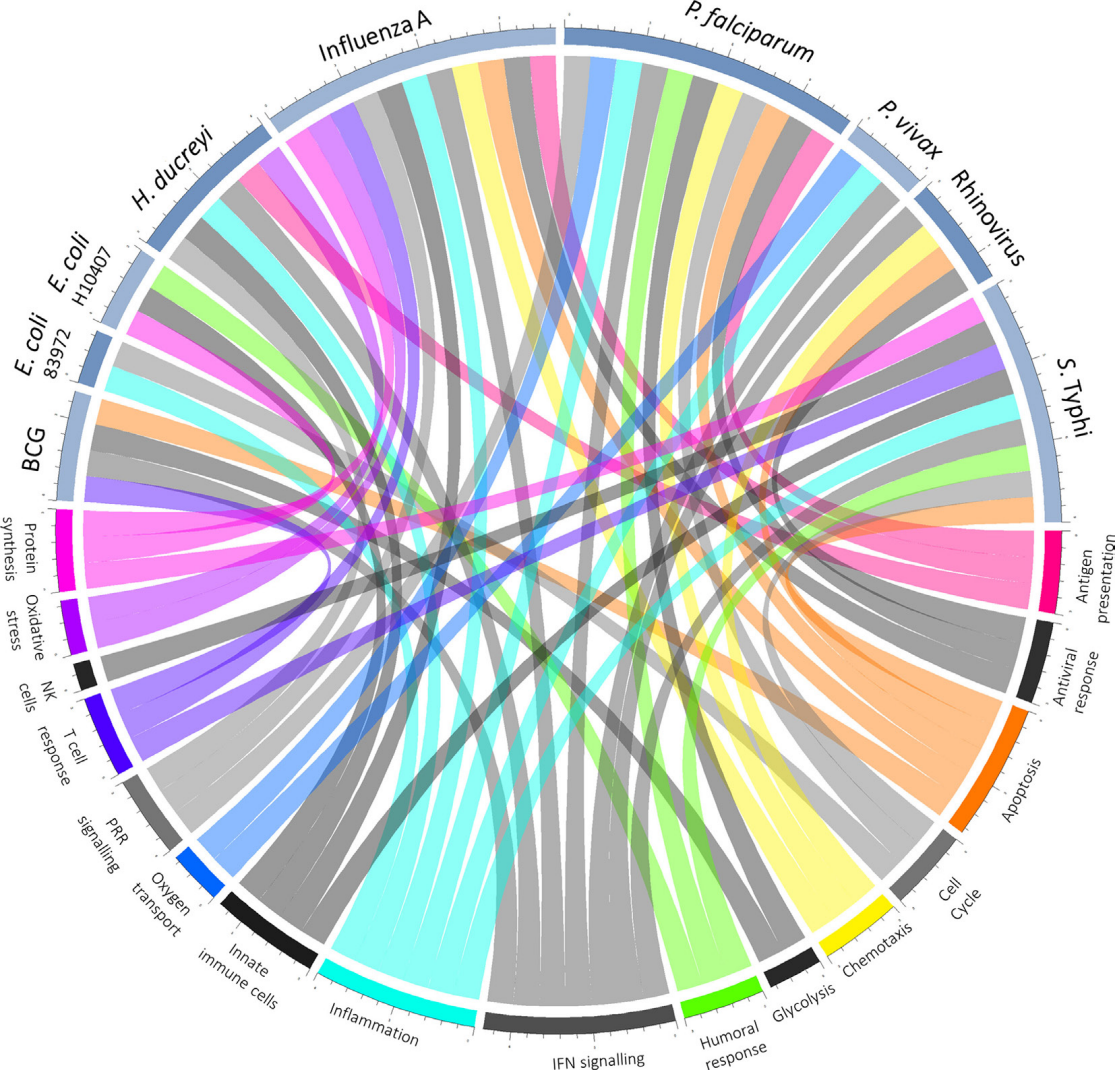
Overview of host–pathogen interactions. Blue arcs encircling the top half of the chord diagram correspond to different challenge agents, while arcs encircling the bottom half represent broad categories of pathways differentially expressed based on the respective transcriptomics study. Where a pathway has been highlighted by the texts as differentially regulated after challenge, a chord joins the agent and pathway.

In those who do become infected, postchallenge transcriptomics can be used to identify pathways previously not known to be involved in disease pathogenesis. An overview of pathways highlighted as differentially regulated after challenge is shown in Figure 2. In addition to reflecting the human response to a pathogen, such pathways may provide insight into pathogen-directed manipulation of the host response. A pathway seemingly ubiquitously upregulated after challenge with intracellular pathogens and parasites is interferon (IFN) signaling, with evidence of induction following challenge with rhinovirus (21), influenza A (22), *Plasmodium falciparum* (23), *Plasmodium vivax* (24), and *Salmonella enterica* serovar Typhi, particularly during periods of detectable bacteremia in typhoid fever (9). By providing a global snapshot of the molecular immune response, transcriptomic data are also well-suited to interrogating response patterns downstream of major immune molecules, as exemplified by studies exploring IFN-γ-induced pathways. By determining which genes had their expression highly correlated with IRF-1 and also contained IRF-1 binding sites in their promoter regions, 12 potential IRF-1 targets upregulated during *P. falciparum* infection were identified (23). Moreover, genes linking IFN-signaling with tryptophan metabolism were found to be upregulated during acute typhoid fever, raising the possibility that IFN-induced tryptophan metabolism plays a role in the host immune response, either by depriving *S*. Typhi of tryptophan or impacting the activation of regulatory T cells (9). Importantly, while transcriptomics provides a cross-sectional profile of the immune response at a given point in time, validation of the results in complementary model systems is pivotal. Thus, multiple validation experiments supported the findings from the typhoid challenge, with the same IFN-γ and tryptophan pathways induced in both a murine model for *Salmonella* infection and *in vitro* infection of human macrophages with *S*. Typhi (9).

As well as confirming the important role of known cornerstones of immunity, analysis of the transcriptional response to pathogenic challenge has highlighted fundamental biological pathways more peripherally associated with immune responses, including apoptosis and the cell cycle. Changes in gene expression indicative of apoptosis take place following experimental infection with rhinovirus (21), *S*. Typhi (9), and influenza A (22), with greater expression of death receptor signaling in those experiencing more severe influenza (25). Furthermore, apoptosis is one of the few pathways overrepresented in natural *P. falciparum* infection compared with experimental infection, possibly as a result of greater parasite load in those naturally infected (23). Another fundamental process often observed in the host transcriptional response to infection is a signature representing the cell cycle, seen to be significantly upregulated following challenge of naïve participants with *S*. Typhi as well as following vaccination (4, 9, 26). While the origin of this signature is unclear, antigen-specific B cell and T cell proliferation for the generation of adaptive immunity is hypothesized to be a factor (4, 9).

Although upregulated pathways are generally those focused upon and pursued in follow-up studies, gene expression changes in both directions during acute infection. One of many drivers downregulating immune components is host manipulation by the pathogen to evade clearance (27). For example, in a small study involving bladder colonization by an asymptomatic strain of *E. coli*, the innate immune response was suppressed, with *in vitro* experiments suggesting that suppression was mediated by inhibition of polymerase II phosphorylation (28). Downregulation of genes in peripheral blood may also represent migration of immune cells to lymphoid tissue and thus changes in the cell composition rather than true expression changes, an important consideration when measuring gene expression in whole blood. Indeed at typhoid diagnosis, transcriptional modules associated with B, T, and natural killer cells are underrepresented (9), possibly reflecting the leukopenia observed in typhoid fever (29) or migration of cells to the intestinal mucosa (16, 30, 31). Moreover, inflammatory genes are downregulated after challenge with *P. vivax* (24), although no change was observed in natural infection with *P. falciparum*. With neutrophil counts accounting for 34% of variance in the inflammation-associated axis (32), this discrepancy might be explained by the neutropenia observed in *P. vivax* but not *P. falciparum* infection (33). Finally, downregulation may represent tolerance by the host to limit immune-mediated damage (22, 34).

Beyond advancing basic scientific understanding, insight into immune pathways elicited by pathogens can facilitate identification of novel therapeutic targets and guide vaccine development toward stimulation of protective immune pathways. The transcriptional changes following ETEC challenge were compared with those induced by a database of small molecule drugs, with a hypothesis that those which correlate closely could be used to augment or modulate antibacterial inflammation (2). This work identified a similar collection of transcriptomic changes for the antibiotic rifampicin, suggesting that the drug might encourage an appropriate immune response in addition to a direct antibacterial effect (2). Thus, by acting as a convenient means to gain an overview of pathogenesis-related pathways, transcriptomics has the potential to identify those amenable to manipulation by drugs. Furthermore, interventions acting on the host response rather than microbial pathways are at a lower risk of evasion through the development of antimicrobial resistance, a factor that will likely be of huge importance in the postantibiotic era.

## Factors Associated with Symptom Severity

The controlled nature, extensive sampling, and close monitoring of participants in human challenge studies provide an invaluable opportunity to correlate changes in gene expression with clinical parameters such as C-reactive protein, temperature, and symptom scores. Expression patterns associated with symptoms are consistently related to innate immunity and inflammation and thus may play an important part in the host response. For example, after rhinovirus challenge expression of the gene *viperin* correlated with rhinorrhea, sneezing, and chills (21). In follow-up experiments on a primary culture of human bronchial epithelium, *viperin* siRNA knockdown increased viral replication, suggesting a protective role. In a recent influenza study aiming to use transcriptomics to predict symptom scores, 16 of the 19 probes identified as predictive were within the antiviral response and innate immunity gene ontogenies (35). Similarly, comparing the transcriptomes of those with mild and more severe laboratory-confirmed influenza demonstrated that signaling pathways involving IFN, pattern recognition receptors, and IRF were significantly enriched in those with moderate/severe disease (25). Moreover, expression of six genes related by IFN signaling distinguished between the two groups with 100% accuracy. During acute typhoid fever, transcriptional modules relating to innate immunity and inflammation were positively correlated with symptom severity and negatively correlated with time to diagnosis (9), suggesting that pathways associated with symptoms are not always protective.

While genes associated with increased symptom scores following malaria challenge have not been directly identified, two studies have compared transcriptomes between groups with differing symptom profiles: experimentally versus naturally infected (23) and naïve versus pre-exposed (24). After challenge with *P. falciparum*, transcriptomes from naïve volunteers, 73% of whom were afebrile at diagnosis due to early detection, were compared with naturally infected febrile participants (23). *CASP-1* and *IL-1*β were upregulated, and IL-1β suppressor *pyrin* downregulated in naturally infected febrile volunteers compared with challenge study participants, suggesting involvement of this inflammatory pathway in fever. In a *P. vivax* challenge study, the transcriptomes of naïve and pre-exposed volunteers were compared to investigate the greater symptom severity in naïve infection, despite equal parasite load and time to diagnosis (24). Many of the transcriptional changes in genes and pathways were more pronounced in the naïve group at diagnosis, suggesting that the pre-exposed group may suffer fewer symptoms due to greater tolerance for the parasite (24).

Whereas many studies focus on gene signatures relating to symptoms, the transcriptional response driving active suppression of symptoms during infection is less well characterized. In an influenza challenge study where gene expression profiles were followed longitudinally in both symptomatic and asymptomatic participants, it was found that asymptomatic participants were indeed successfully infected with influenza, as evidenced by viral shedding, seroconversion, and active perturbation of the transcriptome (22). While upregulated in those with symptoms, genes associated with the inflammasome were downregulated over time in those without symptoms, as were IFN signaling inhibitors *SOCS1* and *SOCS3*. Ribosomal genes associated with lymphocytes, genes which reduce oxidative stress, and Th1-response inducing *SOCS5* were all upregulated. Therefore, the asymptomatic state does not simply represent absence of a pathogen or a response; rather, the subjects appear to mount a regulated immune response, which contains the infection without causing illness. Hypothetically, augmenting these pathways could be used therapeutically to ameliorate symptoms.

Overall, it is not clear which pathways are associated with symptoms as well as protection, which pathways cause symptoms but are non-protective, and which are correlated with, but not causative of, symptomatic disease. Thus, in order to identify targets for the treatment of symptoms, follow-up experiments employing animal and *in vitro* models will be necessary to establish cause-and-effect relationships. For example, in a candidiasis human challenge model polymorphonuclear cell (PMN) infiltration was associated with symptomatic infection (36). A follow-up RNA-seq experiment on mice challenged with *Candida albicans* showed upregulation of phagocyte infiltration and migration pathways, validating observations from the human challenge model (37). In addition, expression of the NLRP3 inflammasome was increased. Although inflammasome inhibition by diabetes drug glibenclamide did not affect colonization in the mice, PMN recruitment was reduced, suggesting that symptoms could feasibly be pharmacologically attenuated. Thus, the use of transcriptomics to identify symptom-associated pathways after human challenge has huge potential to inform therapeutic development.

## Modulation of the Immune Response by Vaccination

Transcriptional responses following human vaccination have been explored extensively, with major studies conducted on vaccines for yellow fever, influenza, and shingles (38–41). Postvaccination challenge studies present the opportunity to rapidly and efficiently test vaccine-mediated protection, as in contrast to Phase III vaccine trials all participants are exposed to the pathogen during the study period. The identification of transcriptomic signatures associated with immunological responses to vaccination (correlates of immunogenicity) and the development of protection (correlates of protection) may facilitate development of vaccines which achieve higher levels of protection against a greater variety of pathogens. Moreover, such signatures could possibly be established as non-serological biomarkers predictive of vaccine-conferred protection (42).

**Figure 3:**
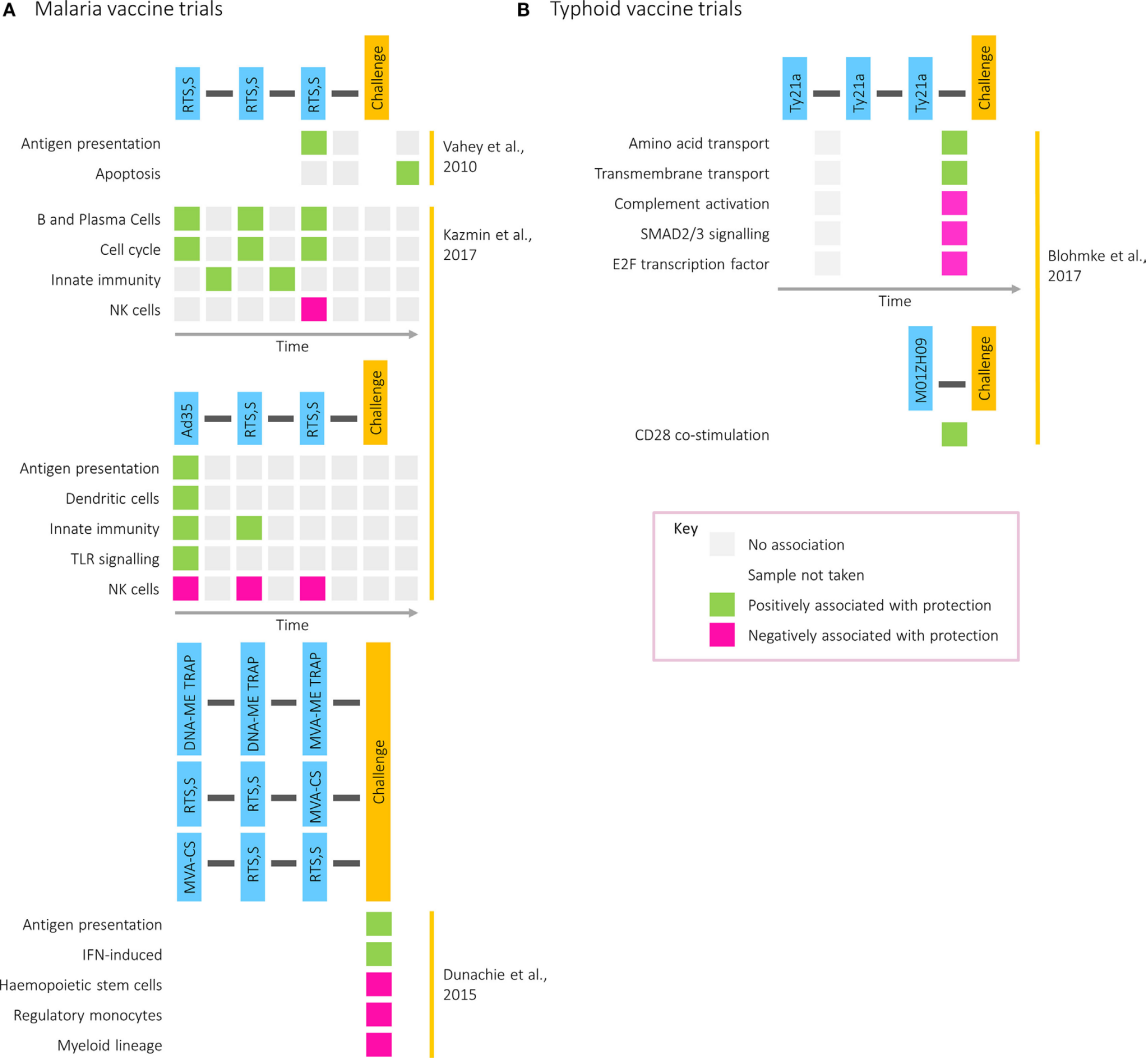
Transcriptional modules associated with protection in **(A)** malaria and **(B)** typhoid postvaccination challenge studies. Rows correspond to pathways or gene modules. Columns correspond to one of the following time points: time of first vaccination, between first and second vaccinations, time of second vaccination, between second and third vaccinations, time of third vaccination, between third vaccination and challenge, time of challenge, and postchallenge. Green squares represent a positive correlation with time to diagnosis and pink squares a negative correlation.

The increasing intricacy of studies combining vaccination and challenge with transcriptomics is demonstrated in work exploring candidate malaria vaccines, which is summarized in Figure 3A. A study comparing two formulations of the RTS,S vaccine identified genes in the immunoproteasome pathway as associated with protection following immunization and were hypothesized to influence immunity through processing of antigenic peptides for presentation on MHC molecules (43). Five years later, a transcriptomic analysis combining two small cohorts receiving different prime-boost vaccine combinations was described, contrasting an RTS,S and a DNA vaccine prime, followed by a boost with two modified vaccinia virus Ankaras expressing different malaria antigens (44). Compared with those who did develop malaria after challenge, the transcriptomes of restimulated PBMCs from the three protected participants showed enrichment of modules associated with IFN induction and antigen presentation. This work illustrates the value of assessing different vaccines together to identify common protective pathways and the advantage of partial vaccine protection for assisting in the identification of protective responses.

Transcriptomics has also been used to investigate factors driving the differences in correlates of protection between two regimens: one where participants received three doses of RTS,S (RRR) and one where participants received the Ad35.CS.01 vaccine followed by two doses of RTS,S (ARR) (4). Although equally protective, antibody titers correlated with protection in the former group and CD4^+^ T cell responses in the latter. Despite largely similar responses at the level of BTMs, with common features including IFN responses at day 1 after vaccination, the BTMs correlating with protection differed between the two regimens. While modules relating to cell cycle, plasma, and B cells after each vaccination correlated with protection in the RRR group, modules relating to DCs and antigen presentation after prime correlated with protection in the ARR group. However, in both groups and at multiple time points, modules related to natural killer cells were negatively associated with protection. Validated on the 2010 RTS,S study data set, the RRR protective signature was capable of predicting whether a participant was protected from malaria with 80% accuracy (4).

More recently, postvaccination transcriptomics has been applied to a typhoid vaccination study, where two oral, live-attenuated vaccines (Ty21a and M01ZH09) were given in conjunction with subsequent pathogenic challenge (45). While NK cell signatures were enriched by Ty21a, the vaccine with the higher protective efficacy, cell cycle modules were strongly induced following vaccination with M01ZH09, inducing higher levels of anti-H and anti-LPS antibodies (46). These data further highlight the association of transcriptional cell cycle markers with humoral responses, as was observed during acute infection (9). Furthermore, whereas gene signatures associated with amino acid and transmembrane transport were correlated with time to diagnosis in Ty21a vaccine recipients, in M01ZH09-vaccinated participants CD28 costimulation was associated with protection (Figure 3B).

Beyond a binary readout of vaccine success, challenge offers the potential to explore vaccine-mediated alterations in the host response, even when complete protection is not achieved. Although identification of differences using this approach is challenging and depends on the vaccine in question (25), both malaria and BCG challenge studies have shown greater changes in global expression patterns in those vaccinated prior to challenge compared with unvaccinated controls (43, 47). These differences can be striking in their magnitude; an over 10-fold difference in the number of differentially regulated genes between vaccinated and naïve participants was observed 5 days postmalaria challenge. Vaccination thus appears to amplify the magnitude of the host response to infection, negatively correlating with mycobacterial growth in the BCG challenge model but positively correlating with scarring at the site of inoculation (47). To conclude, by elucidating key pathways involved in conferring protection, post-challenge transcriptomic studies have the potential to direct rational vaccine development: either by design of antigens which stimulate certain pathways or through the use of adjuvants which enhance protective responses.

## Identification of Diagnostic Biomarkers

Most reliable diagnostic tests for infectious diseases rely on direct detection of a pathogen by culture or PCR. Both methods are labor-intensive and time consuming. Diagnosis on the basis of an antigen-specific host response, however, has been possible for many years, with examples including the tuberculin skin test for tuberculosis (48), the Widal agglutination test for typhoid (49), and ELISA-based antibody assays for dengue (50). Although easy, such tests can have low sensitivity and specificity, are limited in their ability to distinguish between acute infection and previous exposure, and can be confounded by vaccination or immunodeficiency (51).

Recently, transcriptomic analysis has been identified as a useful tool to identify expression signatures specific for a particular condition of interest (52–57). For example, analysis of a gene expression data set derived from a case–control study identified a 27-gene signature discriminatory between acute and latent tuberculosis, regardless of HIV status (57). However, where there is no gold standard for comparison, in the field it is impossible to ascertain the sensitivity and specificity of novel diagnostic tests. The clear and consistent case definitions within human challenge models, and the certainty over with which pathogen a participant is infected, provide a unique opportunity to develop biomarkers and accurately validate diagnostics.

**Figure 4:**
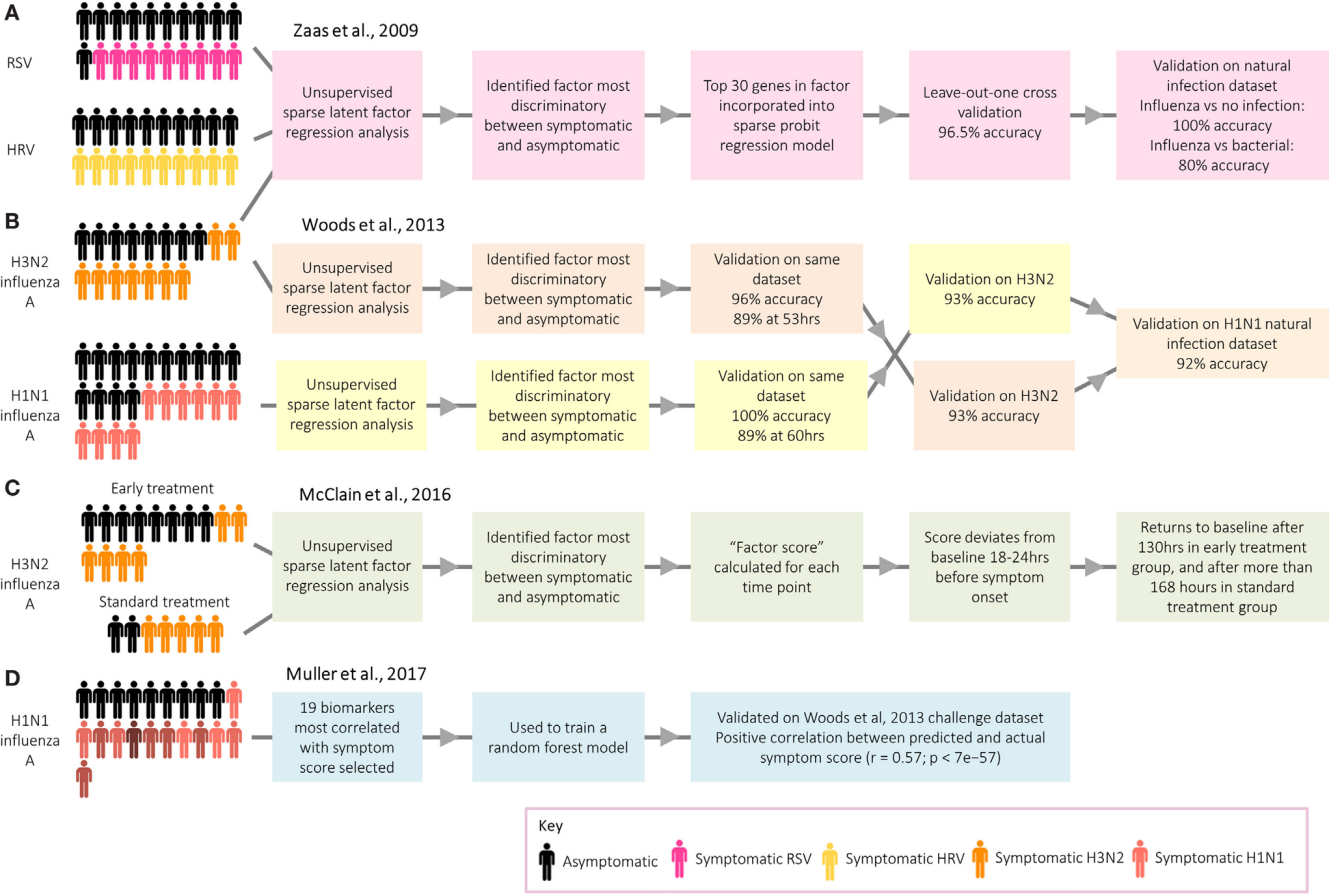
Approaches taken by Duke University and the University of Oxford to identify biomarkers relating to acute respiratory infection. **(A)** Zaas et al. (3) identified a signature for acute respiratory infection. **(B)** Woods et al. (58) identified signatures for influenza infection before peak symptoms. **(C)** McClain et al. (59) compared influenza gene signature score between two treatment groups. **(D)** Muller et al. (35) identified biomarkers predictive of symptom score.

Work by researchers at Duke University on respiratory viral infection has demonstrated the potential use of transcriptomic signatures from challenge models to distinguish between infected and healthy individuals (3). An analysis of microarray data from individuals infected with human rhinovirus, respiratory syncytial virus (RSV), and influenza A identified a group of antiviral response genes which distinguish between symptomatic and asymptomatic participants (Figure 4A). This gene signature predicted whether participants in an independent influenza data set were symptomatic with 100% accuracy. Despite the identification of several genes unique to RSV and human rhinovirus, the signatures for each were similar. More recently, however, transcriptomic analyses of naturally infected patients have identified signatures that can be used to distinguish RSV and influenza from other respiratory infections (54, 56). While challenge studies provide an environment with clear cases useful to identify gene expression signatures indicative of the infection of interest, a limitation is that often these analyses are performed by comparing cases with controls or asymptomatic with symptomatic participants. The question arises whether such signatures can successfully identify a specific infection in a setting where a differential diagnosis with other competing infections has to be reached, in which case the selection of endemic control samples is pivotal for biomarker discovery.

While of limited application in real-world settings, with the exceptions of limiting spread of pandemic infections (58) and detecting individuals exposed to biological warfare agents (60), a series of studies have explored the potential of transcriptomics for presymptomatic diagnosis. Following influenza challenge, factor scores in those developing infection deviated significantly from asymptomatic participants prior to the peak of symptoms (3), with the gene signatures for those developing infection starting to diverge from uninfected participants at 35–40% of the elapsed time between infection and peak symptoms, a point at which symptoms were still very mild [Ref. (58); Figure 4B. The use of these early diagnostic signatures could inform therapeutic interventions which might ultimately influence the outcome of disease. Thus, H3N2 influenza A challenge participants were randomized to either receive early antiviral treatment 36 h postchallenge based on time of divergence in the previous study (58), or “standard” antiviral treatment 120 h after challenge [Ref. (59); Figure 4C. Viral shedding declined quicker in the early treatment group, while symptoms peaked earlier and declined quicker.

Although the main application of transcripts as biomarkers is for diagnosis, a recent challenge study instead aimed to identify a panel of genes which could be used as a method of assessing symptom severity [Ref. (35); Figure 4D]. Distinct from studies focusing on the biological basis of symptoms, discussed earlier in this review, the main aim was to identify biomarkers capable of predicting symptom score. A 19-gene signature was found to be predictive of actual symptom scores, as self-reported by the participants. Such a group of genes could be used to objectively assess the degree to which novel vaccines and therapeutics attenuate symptoms following challenge. Overall, gene signatures have demonstrated great potential as biomarkers for infection and symptom severity, with potential to be used clinically as a convenient and accurate diagnostic tool.

## Strengths and Current Limitations of the Challenge Model Transcriptomics Approach

Controlled human infection models have a number of marked advantages over classical field studies of natural infection, many of which have been alluded to in earlier sections of this review. Here, we examine the strengths and limitations of the application of transcriptomics to challenge studies, which are outlined in Table 1. For a more general discussion of challenge studies readers are directed to a review by Darton et al. (13).

**Table 1:** Strengths and limitations of transcriptomics in human challenge studies.

Strengths	Limitations
The capacity to monitor subjects closely and to collect samples across the time course of infection	Only relatively small sample sizes are feasible

The capacity to obtain preinfection and presymptomatic samples, increasing statistical power through paired samples, and allowing the effect of baseline expression on susceptibility to be examined	Challenge participants are often unrepresentative of populations most affected by a disease, e.g., young adults rather than children or the elderly

Frequent validation on independent challenge and natural infection data sets	Differences in analysis methods and study design can make different studies difficult to compare

Strict selection criteria for study participation, and control of factors such as pathogen dose, strain, and delivery route to minimize variability and reduce required sample sizes	RNA is often extracted from a large number of cell types such as whole blood or tissue homogenate, resulting in averaging and loss of information

The capacity to identify correlates of vaccine-induced protection in small sample sizes	Samples often taken from the peripheral blood rather than sites of infection

Well-defined case definitions	There is often a lack unchallenged controls

The limitation of small sample sizes in challenge studies resulting from practical and cost restraints, in some cases as low as three individuals (28), is pertinent to many types of analysis. Although expression profiling is highly sensitive and capable of detecting small changes between samples from an individual, the power to identify whether such changes are of statistical significance across a group relies on a sufficient sample size. Small numbers are particularly problematic for studies which investigate the link between baseline gene expression and challenge outcome, where high levels of interindividual variation could mask genuine factors affecting susceptibility. Connected with this is the need for appropriate inclusion of non-challenged control subjects, to allow changes unrelated to infection to be accounted for, such as circadian rhythms, variations in sample processing, and random fluctuations in gene expression.

Another major limitation of many of the studies reviewed here is the type of sample available for RNA extraction and profiling. With several exceptions (20, 21), sites of infection such as the intestinal mucosa in enteric infections and respiratory tract in influenza challenges are frequently inaccessible. Furthermore, in the majority of studies RNA is extracted from samples containing a mixed population of cell types (e.g., whole blood). Although pathway and gene set enrichment analyses can identify pathways generally associated with certain cell types, for many pathways it is impossible to tell which cell population is accountable.

An issue common to many transcriptomic studies, and encountered often in their application to human challenge models, is the paucity of validation work and experimental follow-up performed on the genes and pathways highlighted by this approach, either in replicated human experiments, *in vitro* or *in vivo*. Given that challenge participants might not be representative of naturally infected populations, and sample sizes are small, validation on data sets from field studies are critical. For example, although the greatest burden of RSV is in babies, RSV challenge can only be carried out using consenting adults; cohort studies, however, have found that the transcriptomic response to RSV infection changes markedly with age (54). Furthermore, although transcriptomic analyses can identify enriched pathways in response to infection, it is not possible to dissect cause-and-effect relationships from transcriptomic data alone. Identifying which changes fall upstream of others, which are directly related to parameters of interest, and which are merely bystander effects is highly challenging. Therefore, follow-up experiments employing *in vitro* or animal models are extremely valuable in supporting hypotheses, as exemplified by the *in vitro* experiments carried out in the *H. ducreyi* (20), urinary tract *E. coli* (28), and *S*. Typhi (9) challenge studies.

## Changing Technology and Future Directions

While microarrays dominated as the transcriptomic technology of choice in the 2000s as a result of their ease and high throughput, the decreasing cost and increasing sensitivity of next generation sequencing has led to a gradual switch to the use of RNA-seq since 2008 (12, 61). However, only one of the studies reviewed here profiled gene expression by RNA-seq (24), and many of the unique advantages of RNA-seq have not yet been exploited by challenge studies. For example, while microarrays are limited to detecting transcripts represented by the probes on the chip, RNA-seq is capable of detecting non-coding RNAs thought to play an significant role in the antiviral and antibacterial immune responses (62, 63) and thus may act as novel biomarkers or correlates of protection. RNA-seq can also distinguish between different splice variants, of known importance in immunity: for example, in modulating toll-like receptor signaling (64). More recently, differential splicing in Mycobacterium-infected macrophages has been found to produce truncated transcripts that ultimately disrupt macrophage function (65). Thus, in the context of human challenge models, analysis of alternative splicing could give insight into important host–pathogen interactions. Finally, advances in single cell sequencing could be used to characterize transcriptomic changes in different immune cell populations and investigate the heterogeneity within each population. For example, following *ex vivo* infection of macrophages with *Salmonella*, single cell RNA-seq has been used to show that those cells containing growing bacteria possessed M2 expression markers, whereas those containing non-growing bacteria were more pro-inflammatory (66). Thus, the problems associated with averaging expression in the whole blood samples taken in human challenge studies could be eliminated (67).

Although system approaches to immunology have mainly focused on nucleic acid profiling, decreasing costs of mass spectrometry-based technologies such as proteomics (68), metabolomics (69), and lipidomics (70) have resulted in their increased use to characterize infection-induced changes downstream of gene expression. Challenge studies which integrate transcriptomic data with other high parameter data sets will begin to build a more detailed image of the biology of infection and generate a better understanding of how the regulation of gene expression affects functional molecules of the immune system.

Furthermore, the requirement for published transcriptomic data to be deposited in publically available databases has increased opportunities for researchers to compare transcriptomic responses toward a range of pathogens, in various species, and between challenge and natural infection. While one obvious application lies in the identification of specific diagnostic signatures, such approaches stand to deepen our understanding of common and disease-specific elements of immune response.

Although the focus of this review is the human transcriptome, monitoring changes in the transcriptome of the challenge agent itself could substantially benefit our understanding of host–pathogen interactions. For example, using *in vitro* models and clinical samples, changes in *Plasmodium* gene expression during its lifecycle have been characterized by RNA-seq and microarrays (71–73). Selective capture of transcribed sequences (SCOTs), in which bacterial cDNAs are hybridized to biotinylated genomic DNA and captured by streptavidin beads, has emerged as a particularly useful technique for bacterial transcriptomic profiling, as it allows detection of gene expression *in vivo* even at low bacterial titers (74). In the context of human challenge models, this method has successfully been used to identify *H. ducreyi* genes expressed in the pustules of experimentally infected volunteers (75). SCOTs has also facilitated detection of *S*. Typhi and *S*. Paratyphi A transcripts in clinical samples from enteric fever patients (76, 77) and could thus be applied to monitor temporal changes in gene expression in the typhoid human challenge model.

## Concluding Remarks

The application of transcriptomics to human challenge models has given major insight into the postinfection host response, allowing diagnostic biomarkers, correlates of vaccine-induced protection, and indicators of severity to be identified. With increasing availability of data in the public domain and decreasing costs for high-throughput technologies, it is now the time to further investigate host factors associated with susceptibility, exploit the full capabilities of RNA-seq, and integrate transcriptomic data with other “omic” data sets to fully utilize the unique advantages offered by human challenge. It is our ethical obligation to gain as much valuable information as possible from study samples, and transcriptomic profiling is indisputably a key approach which we argue should be considered for application in all future challenge studies. Finally, we would like to emphasize the importance of validation in the field, ensuring that genes or pathways identified as significant are clinically relevant.

## Author Contributions

AB carried out the literature search and created the figures. AB and JH drafted the manuscript. CB, JH, and AP reviewed and made revisions to the manuscript and figures.

## Conflict of Interest Statement

AP has previously conducted clinical trials of vaccines on behalf of Oxford University funded by vaccine manufacturers, but did not receive any personal payments from them. His department received unrestricted educational grants from Pfizer/GSK/Astra Zeneca in July 2016 and from Gilead/MSD/GSK/Astra Zeneca in June 2017 for a course on Infection and Immunity in Children. AP is chair of the UK Department of Health’s (DH) Joint Committee on Vaccination and Immunisation (JCVI), and the scientific advisory group on vaccines of the European Medicines Agency, and a member of WHO’s Strategic Advisory Group of Experts, but the views expressed in this manuscript do not necessarily represent the views of JCVI, DH, EMA, or WHO.
