# Supplementary material for: Transcriptomics in Human Challenge Models
Source: Front Immunol. 2017 Dec 18;8:1839. doi: 10.3389/fimmu.2017.01839 (PMC5741696; doi:10.3389/fimmu.2017.01839)
Supplement: Supplementary file 1 [file Table_1.docx]

Supplementary table 1: Studies exploring transcriptomics in relation to human challenge models. Time points are expressed relative to challenge unless otherwise specified, and only include samples taken for transcriptomic analysis.

| **First author** | **Year** | **Pathogen** | **Sample size** | **Inoculation route** | **Main outcome measures** | **Time points** |
| --- | --- | --- | --- | --- | --- | --- |
| Ockenhouse | 2006 | *P. falciparum* | 22 | Mosquito bite | Blood smear, temperature | 0 hours (hrs), diagnosis |
| Humphreys | 2007 | *H. ducreyi* | 6 | Intradermal | Pustule formation | Day 2 |
| Proud | 2008 | Rhinovirus | 17 18 controls | Intranasal | Viral shedding and seroconversion | Day -14, 8hrs and 2 days after challenge |
| Zaas | 2009 | RSV | 20 | Intranasal | Viral shedding and symptoms | Day -1, 0hrs, then at regular intervals (4-24hrs) for a week |
|  |  | Rhinovirus | 20 |  |  |  |
|  |  | H3N2 Influenza A | 17 |  |  |  |
| Vahey | 2010 | *P. falciparum* | 39 vaccinated 12 non-vaccinated | Mosquito bite | Blood smear | Study entry. Day of final boost and 1 day, 3 days and 2 weeks after. 5 days after challenge. |
| Huang | 2011 | H3N2 Influenza A (see Zaas et. al) | 17 | Intranasal | Viral shedding, symptoms and seroconversion | Day -1, 0hrs, then at regular intervals (8-24hrs) for a week |
| Lutay | 2013 | *E. coli* strain 83972 | 3 | Intravesical | Viable counts in urine, urine PMN numbers and cytokine concentrations | Before challenge, 24hrs |
| Woods | 2013 | H1N1 Influenza A | 24 | Intranasal | Viral shedding and symptoms | Day -1, 0hrs, then at regular intervals (8-24hrs) for a week |
|  |  | H3N2 Influenza A (see Zaas et al.) | 17 |  |  |  |
| Davenport | 2015 | H3N2 Influenza A | 22 | Intranasal | Viral shedding, symptoms, seroconversion | Day -30. 12, 24 and 48hrs after challenge |
| Rojas-Peña | 2015 | *P. vivax* | 16 | Mosquito bite | Blood smear | Day -2, diagnosis |
| Matsumiya | 2015 | BCG | 20 | Intradermal | BCG load by qPCR | Days 0, 2, 7 and 14 |
| Dunachie | 2015 | *P. falciparum* | 6 CSP study 8 TRAP study | Mosquito bite | Blood smear | Day 0 |
| McClain | 2016 | H3N2 Influenza A | 21 | Intranasal | Viral shedding and symptoms | 0hrs, then at 8hr intervals for a week |
| Blohmke | 2016 | *S.* Typhi | 41 | Oral | Fever and blood culture | In diagnosed: 0hrs, 24hrs before diagnosis, diagnosis, 24hrs after diagnosis, 2 weeks after challenge In non-diagnosed: 0hrs, then 1 and 2 weeks after challenge |
| Yang | 2016 | *E. coli* strain H10407 | 12 | Oral | Symptoms and stool output | 8hrs after challenge, peak symptoms |
| Muller | 2017 | H1N1 influenza A | 21 | Intranasal | Symptom score, viral shedding | 0, 24, 48, 72, 96hrs after challenge |
| Kazmin | 2017 | *P. falciparum* | 25 ARR 21 RRR 12 non-vaccinated | Mosquito bite | Blood smear | Day of 1^st^ vaccine and 1, 2 and 6 days after. Day of 2nd vaccine and 1 and 6 days after. Day of 3rd vaccine and 1 and 6 days after. Day of challenge, 1 and 5 days after |
| Blohmke | In press | 99 | *S.* Typhi | Oral | Fever and blood culture | Ty21a: Days -28, -26, -21 before challenge  M01ZH09: Days -24 and -21 before challenge  Placebo: Days -28, -24 and -21 before challenge |
